# Foot Morphology Profile in Indonesian Recreational Runners

**DOI:** 10.7759/cureus.85817

**Published:** 2025-06-11

**Authors:** Astuti Pitarini, Andi Kurniawan, Mitchel Mitchel, Karina S Gani, Jeremy M Sudjaka, Erica Kholinne

**Affiliations:** 1 Orthopedics and Traumatology, Gatam Institute, Eka Hospital, Tangerang, IDN; 2 Orthopedics and Traumatology, St. Carolus Bone and Joint Centre, Jakarta, IDN; 3 Sports Medicine, Eminence Sports Medicine Centre, Jakarta, IDN; 4 Sports Medicine, St. Carolus Sports Clinic, Jakarta, IDN; 5 Orthopedics and Traumatology, Faculty of Medicine, Universitas Katolik Indonesia Atma Jaya, Jakarta, IDN; 6 Orthopedics and Traumatology, Faculty of Medicine, Universitas Trisakti, Jakarta, IDN

**Keywords:** 3d foot scanner, arch index, foot pain, indonesian, recreational runner

## Abstract

Background

Foot morphology is influenced by factors such as physical activity, footwear habits, gender, and body mass index (BMI) and plays an important role in fields like orthopedics and sports science. However, data on the foot morphology of Indonesian recreational runners remain limited. This study aimed to describe the foot morphology of Indonesian recreational runners using a 3D foot scanner.

Methods

A cross-sectional study including 50 recreational runners was included. Data were collected using a self-developed questionnaire and foot scanning using a 3D foot scanner UPOD-S during static standing to assess a few parameters, including BMI, arch index (AI), heel angle, foot length, toe type, shoe size, shoe brand, vitamin D supplementation intake, history, and recent foot and leg pain, which were conducted via a physical examination by a foot and ankle surgeon.

Result

This study included 32 male and 18 female recreational runners aged 22-45 years with a mean BMI of 25.0±7.3 and 24.1±4.1, respectively. The average weekly running distance was 13.8 ± 13.1 km, with participants using various shoe brands. Shoe sizes varied from 36 to 44 (European sizing). Heel angles varied: three feet showed neutral, 20 mild, and one severe angle. All participants exhibited a normal or low AI. Toe types included Roman, Egyptian, and Greek types. The mean foot lengths in males were 256.2 ± 11.9 mm (right) and 256.1 ± 12.0 mm (left), and in females, 229.3 ± 8.6 mm (right) and 229.7 ± 8.7 mm (left). The AI values of most recreational runners were classified as low arch.

Conclusion

Male Indonesian recreational runners generally have longer feet than their female counterparts, although the AI values are similar between the sexes, regardless of foot pain. These mild biomechanical variations may have implications for monitoring and preventing musculoskeletal health issues.

## Introduction

Running is a popular sport and recreational activity worldwide, including in Indonesia. Almost 60% of adults stay active by running, with the number of people participating in organized races increasing by 50% over the past decade [1]. Running is one of the most accessible physical activities, offering significant benefits for improving fitness and preventing obesity, cardiovascular diseases, and other chronic conditions [2]. Despite its surge in popularity, running-related injuries (RRI) and tendinopathy are common, particularly overuse injuries [3]. The injury incidence among short-distance runners (<15 km) ranges from 14.3% to 44.7%, whereas for long-distance runners (half-marathons or marathons), it ranges from 16.7% to 79.3% [4]. Many risk factors contribute to the RRI, including extrinsic factors (such as age, sex, limb mechanics, and genetics) and intrinsic factors (activity level, training factors, loading characteristics, footwear, and medication intake). However, previous research revealed that RRI was the strongest risk factor for injury in long-distance runners. Additionally, significant risk factors for short-distance runners included previous non-RRI (musculoskeletal complaints), higher body mass index (BMI), older age, male sex, lack of running experience, and running less than two hours per week [1].

Human foot morphology is a key focus in physical anatomical studies across various biomedical fields, such as orthopedics, orthotic development, and sports science. Factors such as environmental conditions, daily activities (including the frequency of sports participation and shoe-wearing habits), as well as gender, BMI, and age, have all been shown to significantly influence foot shape and structure in adults [5]. A widely used method for analyzing foot morphology is through 2D footprints, as they are easy to obtain. However, this approach lacks vertical shape information, which limits its accuracy [6]. Foot types such as flat feet or high arches are typically classified based on visual inspection, and numerous footprint-based indices have been developed, primarily targeting the medial longitudinal arch. Despite their practicality, 2D measurements fail to capture the foot's complex 3D structure. Research has shown that attempts to infer 3D morphology from 2D data often result in significant inaccuracies [7].

## Materials and methods

This cross-sectional study included recreational runners participating in a running event. All participants underwent interviews and physical examinations to screen for foot or gait abnormalities. The inclusion criteria consisted of running at least 5 km per week for three consecutive months, either indoors or outdoors, regardless of whether participants were competing in the event. Consent was obtained from each participant. This study employed a simple random sampling approach to select participants and schedule measurements, ensuring unbiased data collection. In this study, we aimed to describe the foot morphology of Indonesian recreational runners using a 3D foot scanner. The parameters evaluated included arch index (AI), heel angle, foot length, and toe type. Additional information, such as BMI, shoe size and brand, vitamin D supplementation intake, and the presence of foot or leg pain, was collected through a combination of physical examination by a foot and ankle surgeon and a self-reported questionnaire. The Research Ethics Committee of Fakultas Kedokteran Universitas Trisakti approved the study (registration number 006/KER/FK/02/2025), and all participants were fully informed of the study's procedures and provided written consent.

Foot morphology

In this study, we measured the AI, heel angle, foot length, and toe type separately for the left and right feet to evaluate foot morphology. The heel angle consists of neutral (0), mild (<4), moderate (4-8), and severe (>8). For the AI, values below 0.21 were classified as high, 0.21-0.26 as normal, and above 0.26 as low. ​​The low category is further subdivided into three levels: low (+) < 0.28; low (++) < 0.30; and low (+++) > 0.30 [8]. The toe type will be classified as either Roman, Greek, or Egyptian. The heel angle was defined as the angle formed between the medial and lateral borders of the posterior heel region. Foot length was defined as the linear distance from the most posterior aspect of the calcaneus to the tip of the longest toe.

Foot morphology data were collected under static standing conditions using a 3D foot scanner UPOD-S (Wuhan City, China) with an accuracy of ±1 mm. The participants needed to follow the steps: (1) Roll up their trousers above the knee and remove their shoes and socks; (2) stand with both feet in a natural posture, eyes and head straight facing front, and place one barefoot (right or left) on the UPOD-S foot scanner within the measuring range; (3) distribute body weight evenly between both feet and maintain balance for three to five seconds until the foot morphology scan is fully recorded; and (4) switch to the other foot and repeat the process.

Pain assessment and self-report survey

A foot and ankle surgeon conducted the foot pain assessments and self-reported surveys of recreational runners following foot morphology measurement. The survey included medical history, physical examination, and personal data (including sex, age, height, weight, email address, and phone number). For foot and leg pain assessment, knee pain was categorized as leg pain, while foot and ankle pain were classified as foot pain. The participants were asked to describe the quality of pain (e.g., burning, stabbing, dull ache), as well as the aggravating and relieving factors, including physical activities (e.g., walking, prolonged standing), footwear, orthotic use, and rest. Clinical examination assessed for associated foot conditions, such as hallux valgus (bunion), calluses, hammer toes, and skin changes.

Additionally, foot pain was divided into forefoot, midfoot, and hindfoot based on anatomical location. The analysis excluded muscle cramps, dermatological conditions, digital calluses, and nighttime paresthesia. Physical examinations were conducted to evaluate the recreational runner's foot and leg pain based on the following steps: (1) The surgeon reviewed the self-reported survey and pain complaints, then guided participants to stand barefoot with their trouser legs rolled up above the knees. (2) The lower extremities were examined through palpation and pressure testing of the feet, ankles, patellae, knees, hips, tibias, fibulas, and femurs, cross-checking the contralateral side for consistency. (3) To precisely localize pain, the surgeon assessed (i) soft tissues: plantar fascia, Achilles tendon, gastrocnemius, tibialis anterior and posterior, biceps femoris, quadriceps, medial/lateral ankle ligaments, anterior cruciate ligament, medial/lateral collateral ligaments, abductor hallucis, abductor digiti minimi, and lower back; (2) bone structures: navicular, cuboid, phalanges, metatarsals, and calcaneus; and (3) joints and key anatomical sites: ankles, patella, knees, hips, and tibia.

## Results

This study involved 50 recreational runners (32 males and 18 females). The participants' ages ranged from 22 to 45 years, with mean body weights of 70.6±11.0 kg for males and 60.1±11.0 kg for females. Shoe sizes varied from 36 to 44 (European sizing). Detailed information is provided in Table 1. The average weekly running distance among participants was 13.8±13.1 km, reflecting considerable variation in training levels.

**Table 1:** Demographic characteristics of the participants Data are given as mean ± SD. BMI: body mass index

Variable	Male	Female
Total patients	32	18
Age (years)	33.1±10.6	35.0±9.9
Body weight (kg)	70.6±11.0	60.1±11.0
Height (cm)	171.7±6.0	157.8±4.7
BMI (kg/m^2^)	25.0±7.3	24.1±4.1
Shoe size	42.4±1.7	38.1±1.5
Regular core training (n)	10	8
Regular aerobic exercise (n)	8	6

Participants wore various running shoe brands, including Hoka, Adidas, Asics, Nike, On Running, Diadora, and New Balance. Seven participants reported regular consumption of vitamin D supplements. Seven left feet and eight right feet exhibited a neutral heel angle, 25 left feet and 27 right feet showed a mild heel angle, and three left feet displayed a severe heel angle (Table 2).

**Table 2:** Heel angle Mild <4; moderate 4-8; severe >8

Sex	Left Foot				Right Foot			
	Neutral	Mild	Moderate	Severe	Neutral	Mild	Moderate	Severe
Male	6	18	6	2	7	17	8	0
Female	1	7	9	1	1	10	7	0

Most participants had normal and low AI in both feet, as detailed in Table 3. The mean AI of the male left foot in those without pain was 0.28±0.04, while in those with pain, it was 0.26±0.04 (mean AI = 0.27±0.04). On the right foot, the AI in males without pain was 0.28±0.03, compared to 0.25±0.04 in those with pain (mean AI = 0.27±0.04). For females, the left foot AI was 0.27±0.02 in individuals without pain and 0.27±0.06 in those with pain (mean AI = 0.27±0.04). Similarly, on the right foot, the AI in females without pain was 0.27±0.03, while in those with pain, it was 0.28±0.06 (mean AI = 0.27±0.04). The details can be seen in Table 4.

**Table 3:** Type of arch index High <0.21; normal 0.21-0.26; low >0.26 Low (+) <0.28; low (++) ≤0.30; low (+++) >0.30

Sex	Left Foot					Right Foot				
	High	Normal	Low			High	Normal	Low		
			+	++	+++			+	++	+++
Male	0	17	1	8	6	1	15	6	6	4
Female	1	9	2	3	3	1	7	2	4	4

**Table 4:** Arch index of the foot in static standing

Variable	Arch Index	
	Without pain	With pain
Left foot		
Male	0.28±0.04	0.26±0.04
Female	0.27±0.02	0.27±0.06
Right foot		
Male	0.28±0.03	0.25±0.04
Female	0.27±0.03	0.28±0.06

The toe types were identified among the participants: Roman, Egyptian, and Greek. In the male group, the mean foot lengths were 257.3±10.3 mm for the right foot and 257.5±11.2 mm for the left foot. Similarly, in the female group, the mean foot lengths were 231.3±8.5 mm for the right foot and 232.5±8.7 mm for the left foot. Foot and leg pain was reported by 21 participants, with the most common pathology being plantar fasciitis, which was found in four participants (8%). Detailed pathology is presented in Table 5.

**Table 5:** Foot and leg pain based on location

Location	n (%)
Forefoot	
Metatarsalgia	3 (6%)
Hallux valgus	1 (2%)
Sesamoiditis	1 (2%)
Midfoot	
Midfoot strain	1 (2%)
Lisfranc injury	1 (2%)
Tibialis posterior tendinitis	3 (6%)
Plantar fibromatosis	1 (2%)
Hindfoot	
Plantar fasciitis	4 (8%)
Achilles tendinitis and/or tendinopathy	2 (4%)
Baxter nerve lesion	1 (2%)
Ankle	
Achilles tendinitis and/or tendinopathy	2 (4%)
Knee	
Patellofemoral instability	1 (2%)

## Discussion

Running is one of the most widely practiced physical activities worldwide. However, although the health benefits of running for physically active individuals have been demonstrated, recreational runners are often affected by RRI at a rate of approximately 79% [4]. Various intrinsic and extrinsic factors, including foot morphology, have been implicated in the development of RRI [1]. However, there is limited data on the specific foot characteristics of runners across different populations. Based on our knowledge, this is the first study that describes the foot morphology in Indonesian recreational runners.

In our recent study, we observed that the AI values of feet with pain and those without pain were nearly identical. This suggests that foot and leg pain do not consistently correlate with variations in foot morphology, particularly when assessed using the AI. Furthermore, arch height variations may not be a key factor in foot pain development. This finding contrasts with previous research linking abnormal AI to foot pain. A possible explanation is that foot pain is multifactorial, involving neuromuscular function, inflammation, and individual pain perception [9]. Additionally, compensation mechanisms such as altered gait patterns or muscle adaptations may help mitigate plantar pressure abnormalities, thereby reducing their direct impact on pain. Esculier et al. reported that gait modifications in runners with patellofemoral pain, such as increasing the step rate by 10% (to 180 steps per minute), decreasing the step rate by 10%, striking with the forefoot or heel, and running softer, are associated with reduced movement-related pain. Additionally, gait modifications may induce analgesic effects that are not solely related to mechanical loading but also involve central pain mechanisms [10].

Previous research has highlighted the utility of the AI derived from footprints as a predictor of foot arch height and a tool for classifying footprint morphology [11]. According to the literature by Cavanagh and Rodgers [8], the AI value for normal arches typically falls within the range of 0.21-0.26. A retrospective study conducted by Chow et al. found the mean AI of both feet in recreational rugby players to be within the normal range. The elite group's arch type fell into the category of low arch, based on 57 male college students (mean age, 20 years). This recent study reported that the elite rugby group more commonly experienced lower limb pain, particularly pain in the metatarsophalangeal joints and the cuboid bone [12]. Our study found that most of the participants were classified as having low-arched feet or flat feet. This finding is consistent with a study by Chow, which reported that Indigenous Taiwanese have lower arched feet compared to non-Indigenous Taiwanese [13].

The data collected in this study show that males generally had longer feet than females on both sides. These gender-based differences in foot morphology align with findings from previous research, which consistently report that men tend to have longer feet than women. Zhao et al. noted that gender has a greater influence on foot dimensions, such as length, width, height, and girth, than BMI or age [14]. Similarly, Castro et al., using calipers and footprint analysis in older Brazilian adults, found that men had significantly higher instep height, forefoot width, and rearfoot width than women [15]. A study utilizing a 3D foot scanner in 291 older adults also confirmed that men exhibited significantly larger values across all major foot dimensions, including length, width, height, and girth, compared to women [16]. Wearing et al. observed that individuals with higher BMIs tend to have a lower plantar arch height. They attributed this finding to fat under the foot, making it difficult to measure arch height [17]. A similar study reported by Domjanic et al. found that a high BMI was associated with wide and flat feet [18]. Kim et al. reported that gender differences in lower limb biomechanics were observed following a 5 km barefoot run. In female runners, there was an increase in loading under the lateral forefoot and lateral midfoot, whereas male runners experienced a decrease [19]. Anatomical variations may influence these differences, as females exhibit greater hip internal rotation, while males show external rotation. Additionally, female runners demonstrated significantly higher hip adduction, hip internal rotation, and knee abduction angles than their male counterparts [20].

In this study, we found that most participants have normal or low-arched feet, which are commonly referred to as flat feet. A study conducted by Powell et al. indicated that individuals with high- or low-arched or flat feet experience a higher incidence of lower extremity injuries than those with normal arches. Furthermore, high-arched feet are associated with an increased risk of bony injuries on the lateral side of the lower extremity. In contrast, low-arched feet are more prone to injuries on the medial side [21]. Low- and normal-arch feet have better shock absorption than high-arch feet. Flat feet result in an uneven distribution of weight across the foot. Over time, this altered weight loading can lead to thickening of the plantar fascia due to increased mechanical stress. Consequently, individuals may rely on excessive knee and hip joint flexion to compensate for limited dorsiflexion during walking, which can ultimately result in joint pain and discomfort [22]. Feger et al. found that subjects with low-arched feet are more prone to an ankle sprain and ankle instability due to weak muscle strength in dorsiflexion and eversion [23]. In marathon and half-marathon runners, toe injuries such as bruised toenails, nail thickening, and subungual hematomas are common. These injuries result from the continuous friction between the toe and the shoe's toe box and can significantly affect a runner's training and performance [24]. Menz [25] also found that the wrong-fitted shoe size was associated with toe corns, hallux valgus deformity, and foot pain.

Marathon running presents a significant challenge for amateur runners. The gait characteristics of marathon runners are crucial, as the feet endure substantial force during running. Research indicates that the prolonged physical exertion associated with marathons can lead to fatigue in the soft tissues supporting the plantar arch, which may, in turn, alter pressure distribution across specific regions of the foot [26]. A study by Toresdahl et al. found that overuse injuries are the primary factors contributing to marathon noncompletion, and strength training did not reduce the incidence of overuse injuries, minor injuries, pain during the race, or the need for medical attention in the marathon medical tent [27]. Foot shape is dynamic and influenced by factors such as obesity and prolonged physical activity, which can result in deformities and variations in morphology. These changes may cause shoes to fit poorly, leading to dermatological issues such as blisters and corns. Additionally, improper footwear can lead to abnormal compensatory movements, thereby increasing the risk of musculoskeletal injuries.

A healthy runner's lifestyle is grounded in balanced nutrition, effective training routines, adequate recovery, and mental well-being. Proper nutrition is essential, emphasizing a diet rich in carbohydrates, proteins, and fats, along with sufficient hydration, to support optimal performance and recovery. Moreover, supplements like vitamin D can play a crucial role in preventing skeletal muscle injuries in athletes after exercise [28]. A similar study reported that lower serum vitamin D levels are associated with increased muscle weakness, fatigue, and a higher incidence of injuries [29]. Furthermore, dietary vitamin D supplementation has been shown to benefit immune system function by reducing exercise-induced proinflammatory cytokines in elite athletes [28]. However, in this study, we did not measure the serum 25(OH)D concentrations due to logistical limitations and the focus of our study.

The primary limitation of our study is the small sample size, which restricts the generalizability of our findings to the broader Indonesian population. This limitation may have reduced the statistical power of our analysis, potentially contributing to the lack of a significant association between the AI and foot and leg pain. Second, although the study focused on foot morphology in recreational runners, we did not provide detailed descriptions of the characteristics related to their running, including duration, intensity, frequency, and warm-up and cool-down protocols. This lack of specificity may hinder reproducibility and limit the ability of clinicians or researchers to implement or compare similar interventions in future studies.

## Conclusions

Male Indonesian recreational runners generally have longer foot lengths compared to females. Interestingly, the AI values in feet with and without pain were remarkably similar between men and women, with most of the genders exhibiting a low arch. This suggests that foot pain in runners with low arches or flat feet is not exclusively attributable to arch structure. As such, clinicians should explore other possible underlying foot conditions during assessment. These findings may suggest a general trend toward mild biomechanical variations in foot morphology among Indonesian recreational runners. Although these differences may seem subclinical, they could hold clinical relevance for orthopedic and sports medicine specialists when designing training programs for runners, as well as for long-term musculoskeletal health monitoring and preventive care.

## Figures and Tables

**Figure 1:**
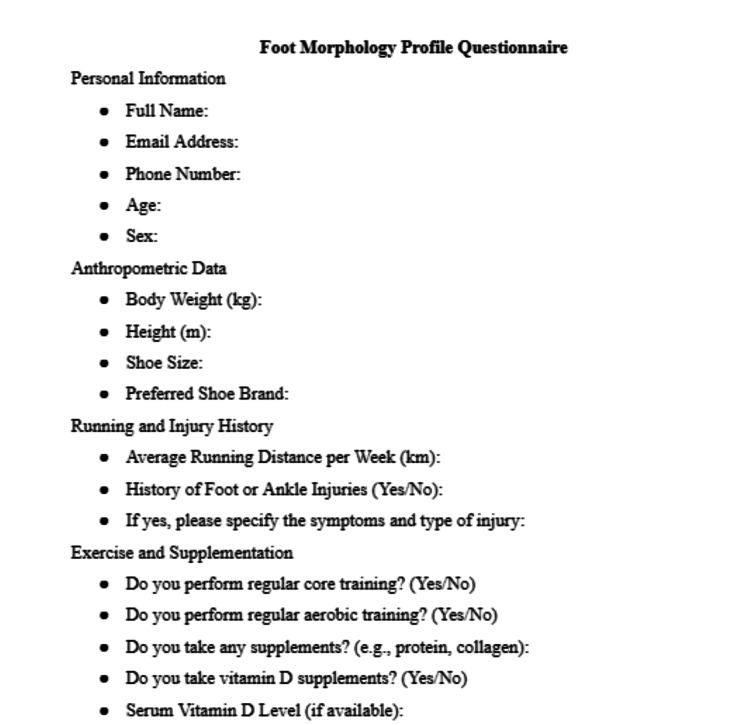
Survey page 1

**Figure 2:**
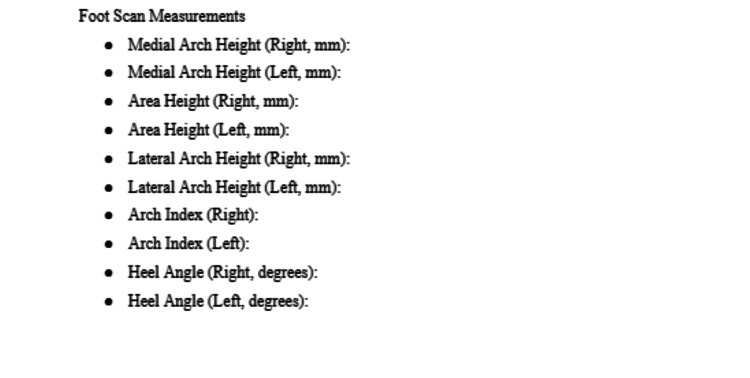
Survey page 2

## References

[1] van Poppel D, van der Worp M, Slabbekoorn A (2021). Risk factors for overuse injuries in short- and long-distance running: a systematic review. J Sport Health Sci.

[2] Dinato RC, Ribeiro AP, Butugan MK, Pereira IL, Onodera AN, Sacco IC (2015). Biomechanical variables and perception of comfort in running shoes with different cushioning technologies. J Sci Med Sport.

[3] Anbarian M, Esmaeili H (2016). Effects of running-induced fatigue on plantar pressure distribution in novice runners with different foot types. Gait Posture.

[4] van der Worp MP, ten Haaf DS, van Cingel R, de Wijer A, Nijhuis-van der Sanden MW, Staal JB (2015). Injuries in runners; a systematic review on risk factors and sex differences. PLoS One.

[5] Stanković K, Booth BG, Danckaers F (2018). Three-dimensional quantitative analysis of healthy foot shape: a proof of concept study. J Foot Ankle Res.

[6] Razeghi M, Batt ME (2002). Foot type classification: a critical review of current methods. Gait Posture.

[7] Luximon A, Goonetilleke RS, Zhang M (2005). 3D foot shape generation from 2D information. Ergonomics.

[8] Cavanagh PR, Rodgers MM (1987). The arch index: a useful measure from footprints. J Biomech.

[9] Hawke F, Burns J (2009). Understanding the nature and mechanism of foot pain. J Foot Ankle Res.

[10] Esculier JF, Bouyer LJ, Roy JS (2022). Running gait modifications can lead to immediate reductions in patellofemoral pain. Front Sports Act Living.

[11] Rosende-Bautista C, Munuera-Martínez PV, Seoane-Pillado T, Reina-Bueno M, Alonso-Tajes F, Pérez-García S, Domínguez-Maldonado G (2021). Relationship of body mass index and footprint morphology to the actual height of the medial longitudinal arch of the foot. Int J Environ Res Public Health.

[12] Chow TH, Chen YS, Hsu CC, Hsu CH (2022). Characteristics of plantar pressure with foot postures and lower limb pain profiles in Taiwanese college elite rugby league athletes. Int J Environ Res Public Health.

[13] Chow TH (2024). Plantar pressure characteristics with foot postures and balance abilities in indigenous Taiwanese: a preliminary exploration. Med Sci Monit.

[14] Zhao X, Tsujimoto T, Kim B, Katayama Y, Tanaka K (2017). Characteristics of foot morphology and their relationship to gender, age, body mass index and bilateral asymmetry in Japanese adults. J Back Musculoskelet Rehabil.

[15] de Castro AP, Rebelatto JR, Aurichio TR (2011). The effect of gender on foot anthropometrics in older people. J Sport Rehabil.

[16] Saghazadeh M, Kitano N, Okura T (2015). Gender differences of foot characteristics in older Japanese adults using a 3D foot scanner. J Foot Ankle Res.

[17] Wearing SC, Hills AP, Byrne NM, Hennig EM, McDonald M (2004). The arch index: a measure of flat or fat feet?. Foot Ankle Int.

[18] Domjanic J, Fieder M, Seidler H, Mitteroecker P (2013). Geometric morphometric footprint analysis of young women. J Foot Ankle Res.

[19] Kim HK, Mirjalili SA, Zhang Y, Xiang L, Gu Y, Fernandez J (2024). Effect of gender and running experience on lower limb biomechanics following 5 km barefoot running. Sports Biomech.

[20] Ferber R, Davis IMC, Williams DS (2003). Gender differences in lower extremity mechanics during running. Clin Biomech.

[21] Powell DW, Williams DS 3rd, Windsor B, Butler RJ, Zhang S (2014). Ankle work and dynamic joint stiffness in high- compared to low-arched athletes during a barefoot running task. Hum Mov Sci.

[22] Park SY, Bang HS, Park DJ (2018). Potential for foot dysfunction and plantar fasciitis according to the shape of the foot arch in young adults. J Exerc Rehabil.

[23] Feger MA, Snell S, Handsfield GG (2016). Diminished foot and ankle muscle volumes in young adults with chronic ankle instability. Orthop J Sports Med.

[24] Zhu C, Song Y, Xu Y, Zhu A, Baker JS, Liu W, Gu Y (2024). Toe box shape of running shoes affects in-shoe foot displacement and deformation: a randomized crossover study. Bioengineering (Basel).

[25] Menz HB (2022). Foot problems. Pathy's Principles and Practice of Geriatric Medicine, Sixth Edition.

[26] Hawrylak A, Matner P, Demidaś A, Barczyk-Pawelec K, Demczuk-Włodarczyk E (2019). Static and dynamic plantar pressure distribution in amateur marathon runners. J Sports Med Phys Fitness.

[27] Toresdahl BG, McElheny K, Metzl J, Ammerman B, Chang B, Kinderknecht J (2020). A randomized study of a strength training program to prevent injuries in runners of the New York City marathon. Sports Health.

[28] Żebrowska A, Sadowska-Krępa E, Stanula A (2020). The effect of vitamin D supplementation on serum total 25(OH) levels and biochemical markers of skeletal muscles in runners. J Int Soc Sports Nutr.

[29] Lewis RM, Redzic M, Thomas DT (2013). The effects of season-long vitamin D supplementation on collegiate swimmers and divers. Int J Sport Nutr Exerc Metab.

